# Comment on: Characterization of the *embB* gene in *Mycobacterium tuberculosis* isolates from Barcelona and rapid detection of main mutations related to ethambutol resistance using a low-density DNA array

**DOI:** 10.1093/jac/dku101

**Published:** 2014-04-20

**Authors:** Claudio U. Köser, Josephine M. Bryant, Iñaki Comas, Silke Feuerriegel, Stefan Niemann, Sebastien Gagneux, Julian Parkhill, Sharon J. Peacock

**Affiliations:** 1 Department of Medicine, University of Cambridge, Cambridge, UK; 2 Wellcome Trust Sanger Institute, Hinxton, UK; 3 Genomics and Health Unit, FISABIO, Valencia, Spain; 4 CIBER (Centros de Investigación Biomédica en Red) in Epidemiology and Public Health, Barcelona, Spain; 5 Molecular Mycobacteriology, Borstel, Germany; 6 German Centre for Infection Research, Research Centre Borstel, Borstel, Germany; 7 Department of Medical Parasitology and Infection Biology, Swiss Tropical and Public Health Institute, Basel, Switzerland; 8 University of Basel, Basel, Switzerland; 9 Clinical Microbiology and Public Health Laboratory, Public Health England, Cambridge, UK; 10 Cambridge University Hospitals NHS Foundation Trust, Cambridge, UK

**Keywords:** *Mycobacterium tuberculosis* complex, phylogenetic diversity, ethambutol resistance

Sir,

We agree with Moure *et al*.^1^ that fast genotypic methods will play an increasingly prominent role in drug susceptibility testing for the *Mycobacterium tuberculosis* complex (MTBC).^2,3^ We would, however, like to point out that the *embB* (*Rv3795*) Glu378Ala polymorphism, which is detected by probe 3 of their newly developed low-density DNA array, is not a marker for ethambutol resistance.^4–7^ Instead, Ala represents the ancestral amino acid at this codon (Figure 1), whereas Glu is present in all modern MTBC (lineages 2, 3 and 4).^6–9^ The MIRU–VNTR data of the 51 ethambutol-resistant isolates from the study by Moure *et al*.^1^ are largely congruent with this finding. All 49 phylogenetically modern MTBC isolates had the *embB* 378 Glu variant. Isolate 5765 was a representative of *Mycobacterium bovis*, which is consistent with the fact that it harboured the Ala variant and was pyrazinamide resistant. By contrast, it was unclear why isolate 233R, which appeared to be *M. bovis* based on its MIRU–VNTR signature, had the Glu variant (experimental error or a homoplastic event might account for this discrepancy).

**Figure 1.**
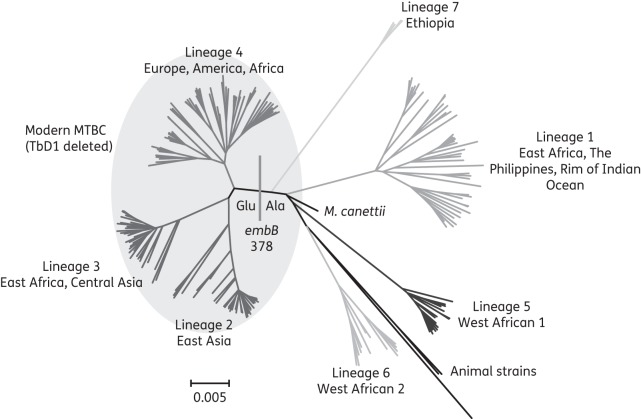
Whole-genome phylogeny of 219 isolates representative of all major MTBC lineages.^9^ Glu at codon 378 is a marker for modern MTBC, which all share the TbD1 deletion and include the lineage 4 *M. tuberculosis* H37Rv laboratory strain that is used as the reference/wild-type sequence for sequence analyses.^10^

In light of these data, the results of probe 3 would be predicted to lead to systematic false-positive reports, which calls into question the validity of this probe. This underlines that the entire MTBC diversity has to be considered when designing and validating genotypic drug susceptibility testing assays.^7,10^

## Funding

This work was supported by a grant from the Department of Health, Wellcome Trust and the Health Innovation Challenge Fund (HICF-T5-342 and WT098600 to S. J. P.), Public Health England (to S. J. P.), the Medical Research Council (to J. M. B.) and the Wellcome Trust Sanger Institute (WT098051 to J. P. and J. M. B). C. U. K. is a Junior Research Fellow at Wolfson College, Cambridge. I. C. is supported by a Ramón y Cajal fellowship from the Spanish Government (RYC-2012-10627).

## Transparency declarations

J. P. has received funding for travel and accommodation from Pacific Biosciences Inc. and Illumina Inc. S. J. P. is a consultant for Pfizer Inc. and has received funding for travel and accommodation from Illumina Inc. All other authors: none to declare.

## Disclaimer

This publication presents independent research supported by the Health Innovation Challenge Fund (HICF-T5-342 and WT098600), a parallel funding partnership between the Department of Health and Wellcome Trust. The views expressed in this publication are those of the authors and not necessarily those of the Department of Health or Wellcome Trust.
